# High Effectiveness of SARS-CoV-2 Vaccines in Reducing COVID-19-Related Deaths in over 75-Year-Olds, Ceará State, Brazil

**DOI:** 10.3390/tropicalmed6030129

**Published:** 2021-07-13

**Authors:** Carlos Henrique Alencar, Luciano Pamplona de Góes Cavalcanti, Magda Moura de Almeida, Patrícia Pereira Lima Barbosa, Kellyn Kessiene de Sousa Cavalcante, Déborah Nunes de Melo, Bruno Cavalcante Fales de Brito Alves, Jorg Heukelbach

**Affiliations:** 1 School of Medicine, Post Graduate Program in Public Health, Federal University of Ceará, Fortaleza 60430-140, Brazil; pamplona.luciano@gmail.com (L.P.G.C.); magda.almeida.mfc@gmail.com (M.M.A.); patricialima18@yahoo.com.br (P.P.L.B.); kellynveterinaria@hotmail.com (K.K.S.C.); deborahnmb@gmail.com (D.N.M.); heukelbach@web.de (J.H.); 2 School of Medicine, Post Graduate Program in Pathology, Federal University of Ceará, Fortaleza 60411-750, Brazil; 3 Christus University Center, Fortaleza 60190-180, Brazil; brunocafa@gmail.com; 4 Health Secretariat of Ceará State, Fortaleza 60060-440, Brazil

**Keywords:** COVID-19, SARS-CoV-2, COVID-19 vaccines, mortality, epidemiology, public health

## Abstract

In Brazil, the SARS-CoV-2 vaccination program has so far prioritized people over 75 years of age. By the end of March 2021, in Ceará State, a total of 313,328 elderly people had received at least one dose of vaccine (45% Oxford-AstraZeneca/Fiocruz and 55% CoronaVac-Sinovac/Butantan), and 159,970 had received two doses (83% CoronaVac-Sinovac/Butantan and 17% Oxford-AstraZeneca/Fiocruz). After a single dose, there was already a significant reduction in COVID 19-related deaths (protection ratio: 19.31 (95% CI: 18.20–20.48), attributable protection ratio: 94.8%); higher protection ratios were observed after the application of two doses of the vaccine (132.67; 95% CI: 109.88–160.18), with an attributable protection ratio of 99.2%. SARS-CoV-2 vaccines are highly effective in reducing the number of COVID-19-related deaths in over 75-year-olds in Brazil, one of the hardest hit countries by the current pandemic.

## 1. Introduction

SARS-CoV-2 vaccines can reduce disease occurrence and transmission in a population. This is essential to reduce both morbidity and mortality from SARS-CoV-2 [1]. Consequently, there is a need for evidence on the effectiveness of vaccines to protect not only against SARS-CoV-2 symptoms but also to reduce COVID-19-related case fatality rates [2]. However, the reduction in the occurrence of severe disease and death is difficult to evaluate in phase 3 clinical trials, mainly due to the high number of participants required [1]. Thus, the effectiveness of SARS-CoV-2 vaccines in relation to case fatality has to be inferred from other sources of data, such as mortality statistics [3].

In Brazil, by the end of June 2021, more than 18.5 million cases and more than 500,000 deaths were confirmed, with a case fatality rate of 2.8% [4]. The state of Ceará, with a population of 8.8 million, was one of the first Brazilian states to confirm sustained transmission of COVID-19 in 2020 [5]. Despite the rapid implementation of control measures, Ceará stands out with more than 880,000 cases and almost 22,500 deaths by the end of June 2021 [4]. The case fatality rate was 2.5%, and there was a high rate of hospital bed occupancy (>90%), while different strains of SARS-CoV-2 were circulating [5].

A recent case-control study in England, including almost 160,000 adults aged over 70 years, evidenced a significant reduction in symptomatic COVID-19 cases and severe symptoms after a single dose of the Oxford-AstraZeneca vaccine [6]. A recent study with Brazilian data showed an association between the rapid increase in vaccination coverage of the older population and relative mortality, as compared to younger individuals, in a setting where the gamma variant was predominant, and the most widely used vaccine was CoronaVac-Sinovac/Butantan [7]. The Brazilian Ministry of Health has made available both vaccines from Oxford-AstraZeneca/Fiocruz and CoronaVac-Sinovac/Butantan. In Ceará, by May 2021, more than 1.7 million people had taken at least one dose of a vaccine, with more than 500 thousand people having received two doses [8]. In this study, we evaluated the hypothesis that COVID-19 vaccinations had a considerable impact on reducing the number of deaths due to COVID-19 in the state of Ceará, Northeast Brazil, in the year 2021.

## 2. Materials and Methods

People aged 75 years or older were included since this age group was prioritized by the Brazilian Immunization Program and, thus, had a higher proportion of vaccination coverage at the beginning of the campaign. For the year 2021, the estimated population in this age group was 354,269 people (IBGE—Brazilian Institute of Geography and Statistics/*Instituto Brasileiro de Geografia e Estatística*). 

We used data from the National Mortality System (SIM) and from the Immunization Program (SIPNI), between 17 January and 11 May 2021. The SIM database records all deaths that occur in Brazil. We selected death records with COVID-19 as the underlying cause of death. The SIPNI aims to coordinate immunization actions throughout Brazil, and records the immunobiological doses applied. The number of unvaccinated people was calculated as the difference between the estimated population and the number of vaccinated individuals. We included only individuals who had received at least one COVID-19 vaccine application. After removing duplicates, the databases were probabilistically related by means of people’s names (*soundex*) and respective dates of birth, using Stata 15.1 software. The outcome was defined as people who died 21 days or later after the first dose of vaccine. We stratified the vaccinated population by number of doses, vaccine type and age group, and calculated the proportion of deaths as well as the protection ratio for deaths and percentage attributable protection ratio for deaths, and their respective 95% confidence intervals. All data in this study were extracted from secondary databases. The use of data was authorized by the Secretary of Health of the State of Ceará. As the study consisted of an analysis of secondary data, no informed consent was sought. 

## 3. Results

A total of 313,328 elderly people (88.4% of the total population > 75 years) had received at least one dose of a vaccine, 44.5% from Oxford-AstraZeneca/Fiocruz and 55.5% from CoronaVac. A total of 159,970 had received two doses, 83.0% from CoronaVac-Sinovac/Butantan and 17.0% from Oxford-AstraZeneca/Fiocruz. The occurrence of deaths among the unvaccinated elderly was more than 132 times higher, as compared to those who had received two doses of a vaccine, with a protection ratio for deaths of 99.2%. After a single dose of a vaccine, the protection ratio was 19.3 (Table 1). The effect was more pronounced with increasing age. 

## 4. Discussion

Our data showed an impressive reduction in COVID-19-related deaths in older age groups in Ceará State, which is the population strata at highest risk for severe disease and death. Previous studies have shown that, by May 2021, more than 40,000 deaths had been prevented due to vaccination of the elderly population in Brazil with the Oxford-AstraZeneca and CoronaVac-Sinovac/Butantan vaccines [7]. Similar findings were found in the US after use of the first dose of Pfizer-BioNTech, particularly in older adults [9]. A study in Tennessee/USA showed a reduction of more than 95% in mortality in the vaccinated elderly population between December 2020 and March 2021 [10].

Considering the difficulties in the vaccine supply chain and their availability, it is important that the vaccines from both major producers showed a high effectiveness in reducing COVID-19-related deaths, even after a single dose. Furthermore, as predicted by Bolcato et al. in 2020, there may be problems that occur, such as insufficient production of vaccine doses for the entire population, or with different vaccination strategies and different times between doses, generating the need for difficult prioritization decisions [11]. In this context, the ability of a vaccine to protect against serious illness and death should be considered the most important outcome, since hospital admissions, especially in intensive care units, represent the greatest burden on health systems and has led several countries to face a collapse in their health systems. The global crisis generated by the coronavirus pandemic highlighted, once again, the importance of vaccination programs as effective public health measures, and brought about new mechanisms that may become models for future responses to regional epidemics and pandemics, with a greater variety of platforms and joint work to overcome challenges and accelerate vaccine development, manufacturing and delivery [12]. It is worth noting that the duration of protection after recovery from COVID-19 corresponds somewhat to the duration of protection provided by the vaccine [13]. 

For Hodgson et al. (2021), the beneficial effects of a vaccine can be assessed if the vaccine is effective in older adults and if there is a wide distribution of the vaccine [1]. The evaluation of asymptomatic SARS-CoV-2 infection is an important clinical outcome in the evaluation of vaccines, but is certainly of less public health importance than its effectiveness against death. In Italy, for example, the number of infections in nursing homes was particularly high, with a high mortality rate. Yet it must be recognized that the current situation of social disparity does not facilitate equal opportunities for all. As a result, the elderly will continue to experience moments of loneliness, despite efforts to reduce them [14].

Equal access to COVID-19 vaccines in all countries will continue to be a goal to be pursued. But the experience of previous pandemics suggests that access will be limited in low and middle income countries, despite the rapid development of some new candidate vaccines. Thus, the WHO proposal, with the COVAX Facility program, represents an attempt to facilitate multilateral cooperation to procure and distribute two billion doses of COVID-19 vaccines equitably in all countries of the world by the end of 2021 [15].

Our study is subject to some limitations, such as the use of secondary mortality data that may be subject to some errors. The smaller number of second doses by AstraZeneca in our study is basically due to the longer period between the two doses and, therefore, the population had not yet received the second dose during the study period. We also observed that the population of people vaccinated in the age group over 90 years was higher than the estimated population for this age group, this fact is due to the last census being conducted in 2010. We used the population projection for the year 2021, but there was still a difference of 1900 more people vaccinated in the population over 90 years of age. The estimated population was adjusted to the vaccinated population and, thus, data should be interpreted with care. Data on the antibody response of vaccinated individuals were not available, which may limit interpretation of results. However, we obtained population-based data from a population with a high vaccination coverage, and the study results can, thus, be considered as robust and valid. 

## 5. Conclusions

SARS-CoV-2 vaccines are highly effective in reducing the number of COVID-19-related deaths in over 75 year-olds in Brazil, one of the hardest hit countries by the current pandemic. 

## Figures and Tables

**Table 1:** Protection ratios for death and percentage attributable protection ratios for deaths by COVID-19, stratified by number of doses applied, vaccine type and age group over 75 year-olds in the state of Ceará, Brazil, 2021.

Variables	N	Deaths	% Deaths	Protection Ratio (95% CI)	Attributable Protection Ratio (%) (95%CI)
Number of doses and type of vaccine:					
Oxford-AstraZeneca/Fiocruz 1st dose	139,322	716	0.51	17.91 (16.55–19.39)	94.4 (93.9–94.8)
CoronaVac-Sinovac/Butantan 1st dose	174,006	778	0.45	20.59 (19.07–22.22)	95.1 (94.7–95.5)
Vaccinated 1st dose	313,328	1494	0.48	19.31 (18.20–20.48)	94.8 (94.5–95.1)
Oxford-AstraZeneca/Fiocruz 1st and 2nd dose	27,193	3	0.01	834.45 (269.03–2588.18)	99.8 (99.6–99.9)
CoronaVac-Sinovac/Butantan 1st and 2nd dose	132,777	108	0.08	113.17 (93.50–136.99)	99.1 (98.9–99.3)
Vaccinated 1st and 2nd dose	159,970	111	0.07	132.67 (109.88–160.18	99.2 (99.1–99.4)
Not vaccinated	40,941	3769	9.21	1	-
Age Group–1st dose only:					
75 to 79 years					
Oxford-AstraZeneca/Fiocruz	32,749	141	0.43	8.39 (7.03–10.00)	88.0 (85.8–90.0)
CoronaVac-Sinovac/Butantan	97,072	481	0.50	7.29 (6.54–8.12)	86.3 (84.7–87.7)
Vaccinated	129,821	622	0.48	7.53 (6.82–8.33)	86.7 (85.3–88.0)
Not vaccinated	26,857	1010	3.76	1	-
80 to 89 years					
Oxford-AstraZeneca/Fiocruz	78,474	371	0.47	31.89 (28.59–35.58)	96.8 (96.5–97.2)
CoronaVac-Sinovac/Butantan	70,327	256	0.36	41.42 (36.42–47.12)	97.6 (97.2–97.9)
Vaccinated	148,801	627	0.42	35.78 (32.77–39.07)	97.2 (96.9–97.4)
Not vaccinated	13,336	2011	15.08	1	-
90 years or more					
Oxford-AstraZeneca/Fiocruz	28,099	204	0.73	137.74 (120.13–157.92)	99.2 (99.1–99.4)
CoronaVac-Sinovac/Butantan	6,607	41	0.62	161.14 (118.76–218.64)	99.3 (99.1–99.5)
Vaccinated	34,706	245	0.71	141.65 (125.04–160.48)	99.3 (99.2–99.4)
Not vaccinated	748	748	100.00	1	-

## Data Availability

The data presented in this study are publicly available in: https://integrasus.saude.ce.gov.br/#/indicadores/indicadores-coronavirus/indice-transparencia (accessed on 13 July 2021).
